# Characteristics of Seltorexant—Innovative Agent Targeting Orexin System for the Treatment of Depression and Anxiety

**DOI:** 10.3390/molecules28083575

**Published:** 2023-04-19

**Authors:** Wojciech Ziemichód, Antonina Kurowska, Karolina Grabowska, Michalina Kurowska, Grażyna Biała

**Affiliations:** 1 Chair and Department of Pharmacology and Pharmacodynamics, Medical University of Lublin, 4a Chodźki Street, 20-093 Lublin, Poland; 2 Institute of Applied Psychology, Jagiellonian University, 31-007 Warsaw, Poland

**Keywords:** orexin system, depression, anxiety, seltorexant

## Abstract

Twenty-five years have passed since the discovery of the orexin system, during which time we have learned more and more about it. A number of studies have been conducted showing the role of the orexin system in insomnia, as well as its potential use in the treatment of obesity and depression. In this review, we present the role of the orexin system in the development of depressive illness and show the characteristics of seltorexant, a potential drug for the treatment of depression. This review describes the structure and synthesis of the compound as well as its pharmacodynamics and pharmacokinetics. Pre-clinical and clinical studies are also described, including side effects. There is evidence that the use of seltorexant is considered safe, with no clear or major clinically significant side effects, which makes it a promising candidate for the treatment of depression and anxiety disorders.

## 1. Objective of the Work

The aim of this study was to present the relationship between the disturbed functioning of the orexin system and the occurrence of mental conditions. We also intended to provide an extensive review of the literature data on seltorexant, which is a selective orexin receptor antagonist. Data from the last 25 years (1996–2023) were collected for the preparation of this manuscript. The literature review was conducted using databases such as those on the pubmed.com, scopus.com, and pubchem.com websites. Access to some data was made possible thanks to the main library of the Medical University in Lublin.

## 2. Introduction

The orexin system was discovered in 1998, and it comprises two endogenous peptide ligands for two orphan G protein-coupled receptors (GPCRs). Initially, they were identified as feeding behavior regulators. The neuropeptides orexin-A (hypocretin-1) and orexin-B (hypocretin-2) are produced by a cluster of neurons in the lateral hypothalamus and perifornical area, which suggests their crucial role in the maintenance of homeostasis [1].

Orexin neurons receive and transmit signals related to environmental, physiological, and emotional stimuli throughout the central nervous system (CNS). The activity of orexins is controlled by their specific receptors (OX1R, OX2R). OX1R is found in the prefrontal and infralimbic cortex (IL), the hippocampus (CA2), the amygdala, the stria terminalis bed nucleus (BST), the paraventricular nucleus of the thalamus (PVT), the anterior hypothalamus, the dorsal raphe (DR), the ventral tegmental area (VTA), the locus coeruleus (LC), and the laterodorsal tegmental (PPT). The dorsomedial hypothalamic nucleus (DMH), the paraventricular nucleus (PVN), the lateral hypothalamic area (LHA), BST, PVT, DR, VTA, the laterodorsal tegmentum (LDT), the pedunculopontine tegmental nucleus (PPT), CA3 in the hippocampus, and the medial septal nucleus all contain OX2R [2,3]. Recent research on the efferent and afferent systems of orexin-producing neurons has suggested that, in addition to regulating feeding patterns, orexin may play important roles in the coordination of emotion, energy homeostasis, reward, and arousal. To maintain a long consolidated waking period, orexin activates monoaminergic and cholinergic neurons in the hypothalamus and brainstem regions. The responsiveness of orexin neurons to peripheral metabolic cues such as leptin and glucose suggests that these neurons play a crucial role in linking energy homeostasis and vigilance states. In animal models, using a model of transgenic mice, orexin deficiency led to obesity even when calorie consumption was lower than in wild-type counterparts [4]. Orexin signaling also promotes obesity resistance through increased spontaneous physical activity and energy expenditure regulation [4,5,6]. The dopaminergic reward system in the VTA is also linked to orexin neurons. To maintain proper vigilance states, the orexin system appears to interact with neuronal pathways that regulate emotion, reward, and energy homeostasis. As a result, this system may be a promising therapeutic target for the treatment of sleep disorders, obesity, emotional stress, and addiction (Tsujino and Sakurai, 2009). These findings suggest that orexin neurons are involved in sensing the body’s external and internal environments and regulating sleep and wake states as needed for survival [3]. 

Interestingly, the orexin pathway has emerged as a critical component in maintaining awakeness and vigilance. The hypocretin system is highly conserved among vertebrates; it is necessary for arousal and alertness stability and cannot be replaced by another brain circuit. The discovery of hypocretin/orexin quickly led to the theory that it had a pathogenic role in the abnormal state of hyperarousal in insomnia, as well as narcolepsy and cataplexy in its shortage. A selective loss of orexin neurons was reported in narcolepsy, confirming the importance of orexins in maintaining wakefulness [3].

As shown above, the dysfunction of the orexinergic system may underlie a variety of pathological conditions. Other roles of orexins, aside from regulating wakefulness, are emerging, such as the modulation of stress-, feeding-, and reward-related circuits within the brain. The orexin system, with its two distinct peptides and two receptors that are variably released and distributed throughout the brain, contributes significantly to this functional variety. 

The effects of dual orexin receptor antagonists (DORA) have been described by various research centers. The two DORAs, suvorexant and lemborexant, are approved by the Food and Drug Administration (FDA) for the treatment of primary insomnia. A third molecule, daridorexant, that improves not only sleep but also daytime functioning in patients with insomnia is under review for approval by health authorities. These three DORAs are all efficacious in reducing latency to sleep and wake after sleep onset, and they prolong total sleep time without altering the physiological sleep architecture [7,8,9].

The first clinical trials on selective orexin 1 or 2 receptor antagonists (SORAs) have begun, and they are being studied for the treatment of mood, anxiety, and eating disorders. Orexin receptor agonists are being researched as potential new therapies for narcolepsy [1] as recent research indicates that the loss of lateral hypothalamic neurons, which produce the neurotransmitter hypocretin, is the primary pathophysiology of human narcolepsy. Hypocretin ligand deficiency affects approximately 90% of people with narcolepsy and cataplexy [10]. Experiments with hypocretin cell-ablated mice showed that the central administration of hypocretin-1 reduces cataplexy and increases waking time in these animals. This suggests that, at least in this paradigm, the loss of the hypocretin peptide does not result in a permanent loss of ability to respond to hypocretin-1 in a physiologically meaningful manner, and that such a response can be elicited without the use of a spatially targeted medication [11].

Hypothalamic orexin neurons project extensively to stress-sensitive brain areas such as the amygdala, BNST, and mPFC. The antagonism of orexin receptors appears to be a very promising therapeutic pathway for the treatment of psychiatric diseases. Given the orexin system’s significance in stress reactivity and fear reactions, orexin-related pharmaceutical targets may represent interesting avenues for treating depression and anxiety. SORA1s have been shown to reliably attenuate fear and anxiety-like behaviors in rodents. More clinical trials are needed to assess their efficacy in people suffering from anxiety and panic disorders [12].

According to The Institute of Health Metrics and Evaluation, an independent global health research center, depression affects approximately 280 million people in the world. It is a common condition, with an estimated 3.8% of the world’s population affected. The Global Burden of Disease Study conducted by the WHO ranked depression as the single most burdensome condition in the world in terms of total disability-adjusted life years among people in midlife [13]. Depressive episodes vary in intensity from low to severe and might become a serious health condition. It carries economic costs in the hundreds of billions and is a major risk factor for suicide. Accessible and effective treatment for such a complex disorder as depression is crucial to improving the well-being of humanity.

Depressive disorders are mainly characterized by a depressive mood (e.g., feeling sad, empty, hopeless) or loss of pleasure (anhedonia). According to the eleventh revision of the International Classification of Diseases, to identify depression, the aforementioned manifestations must occur for most of the day, almost every day over a period of at least two weeks, accompanied by other symptoms such as difficulty concentrating, feelings of worthlessness or excessive or inappropriate guilt, hopelessness, recurrent thoughts of death or suicide, changes in appetite or sleep, psychomotor agitation or retardation, and decreased energy or fatigue.

The diagnosis of depression is preceded by an in-depth interview, in which the listed symptoms must be found. Tests such as Beck’s Depression Inventory are used for initial diagnosis [14]. This scale contains statements assessing the respondent’s attitude toward themselves and the world around them, quality of their sleep and appetite, perceived satisfaction or guilt, and many more. The respondent selects the 21 most fitting statements, which are scored differently. Scores above 21 indicate moderate depression.

Determining the etiology of depression is a difficult task and to this day, research on this topic is ongoing. A recent study has shown that despite a dominant narrative, depression is not caused by serotonin abnormalities and there is no convincing scientific evidence to support that statement [15]. Many people take antidepressants believing their depression has a biochemical cause. A 2010 review of the literature suggests that antidepressants are only marginally efficacious compared to placebos and documents profound publication bias that inflates their apparent efficacy, according to the FDA, indicating the need for a reappraisal of the current standard care for depressive disorders [16]. In light of these discoveries, there is a need to seek new treatments tailored to the complexity of the factors that cause mood disorders.

## 3. Orexin and Its Influence on Depression and Anxiety

As depression is a growing health problem, scientists are investing a lot of effort in understanding this multifactorial disease and finding new targets for its treatment.

Research indicates that dysregulated orexin signaling has been observed in various neuropsychiatric disease states such as depression and anxiety [17]. As was confirmed, hypothalamic orexin neurons have extensive projections to stress-sensitive brain regions including the amygdala, bed nucleus of the striaterminalis (BNST), and medial prefrontal cortex (mPFC) [12,17]. Furthermore, acute restraint stress activates orexin neurons in the lateral hypothalamus (LH) and increases the levels of orexin-A in the VTA [12,18]. These changes cause abnormal behavioral stress reactivity and reduced motivated arousal, which are compelling symptoms of depression [17]. Studies also indicate that a high orexin-A level is related to panic anxiety. However, it was shown that when panic anxiety was comorbid with major depressive disorder (MDD), orexin-A levels were lower than in the group with only panic anxiety [19].

James and co-workers [20] conducted an evaluation of the changes in orexin system function and its influence on behavioral phenotype in adulthood caused by early life stress (ELS)-induced depression. Wistar rats (both males and females) were subjected to maternal separation stress on postnatal days 2–14. Subsequently, a group of animals was given access to running wheels in late adolescence (one hour a day, 40–70 h after birth). Adult rats were exposed to restraint stress and then tested on the open field (OF) as well as elevated plus maze (EPM). After this time, their brains were processed for FOS-protein, orexin, and tyrosine hydroxylase immunohistochemistry. As a result, scientists stated that restraint stress stimulated FOS-protein expression in peripheral area orexin cells, paraventricular hypothalamic nuclei, and paraventricular thalamic nuclei. Interestingly, this neuronal response was dampened in male and female rats exposed to early life stress. Additionally, ELS reduced exploration in OF with no influence on EPM behavior, which is consistent with a depressive-like phenotype. It is noteworthy that other research indicated reduced motivation to press a lever for sucrose (Cambell et al., 2014). What is most intriguing, however, is that adolescent exercise reversed orexin and behavioral deficits in ELS males but not in females. In conclusion, scientists have shown that the orexin system is plastic [20]. Evaluation of Wistar Kyoto rats, which exhibit behavioral and hormonal profiles similar to those observed in patients with diagnosed depression (e.g., increased behavioral despair, altered corticosterone responses to psychological stressors) [21] revealed that these rats have a wide range of orexin-related deficiencies. Kyoto rats have lower numbers of hypothalamic orexin neurons, reduced orexin somasize, reduced levels of prepro-orexin mRNA levels and lower orexin-A, immunoreactivity in various regions including the hypothalamus and amygdala [17,22,23].

Additionally, rats that are chronically housed under dim light conditions (the seasonal affective disorder model) presented increased immobility in the forced swim test as well as reduced sucrose preference [24]. Furthermore, both mice and rats exposed to chronic social defeat stress exhibit increased depressive-like behavior and have decreased expression of orexin mRNA, reduced orexin cell numbers, and elevated levels of both orexin-A and orexin-B peptides. Depressive-like behavior can be reversed using ICV infusion of orexin-A, as confirmed in [25].

Surprisingly, not all of the preclinical studies elucidating depression model behavior report the downregulation of orexin system function [17]. Mikrouli and colleagues proved that the Flinders Sensitive Line rat, which is a genetic model of depression, has increased numbers of orexin neurons [17,26]. This corresponds with the evaluation of Cengiz and co-workers, who investigated whether the orexin receptor 1 and 2 (ORX1, ORX2) genes were associated with MDD development. In this trial, 75 patients with MDD and 87 healthy subjects were enrolled. Genotyping using real-time polymerase chain reaction (RT-PCR) revealed that there is a significant relationship between the genotype frequencies of ORX1 between MDD patients and the control group. As a result, ORX1 (rs10914456 and rs2271933) can be associated with MDD and may affect depressive symptom severity. Interestingly, there was no association between the Orx2 rs2653349 genotypes and MDD development [27].

Brundin et al. evaluated the orexin levels in the cerebrospinal fluid (CSF) of 66 suicidal patients with MDD, dysthymia, and adjustment disorder in their human research on the relationship between depression and the orexin system. It is noteworthy that blood samples from patients were free from antidepressive and neuroleptic medication at the time of the lumbar punctures. As a result, scientists discovered that the CSF levels of orexin-A in patients with MDD were significantly lower than in patients with dysthymia and adjustment disorder. Orexin levels were significantly correlated with peptides involved in the regulation of sleep and appetite such as somatostatin, delta sleep-inducing peptide-like immunoreactivity, and corticotropin releasing factor, whereas they were not with leptin or vasopressin [28]. The same research group extended their evaluation to measure the levels of orexin in the cerebrospinal fluid in 10 patients who took part in lumbar punctures as well as psychiatric evaluation in conjunction with a suicide attempt and after 6 and 12 months. It transpired that the SCF-orexin level was significantly higher 6 months and 12 months after the suicide attempt (orexin level was higher in all patients) [29].

As the role of orexins in the development of MDD is complex and remains puzzling and unclear, there is a large scope for further evaluation. It is significant that there are some efforts to treat depression and panic disorders by affecting the orexin system. There is evidence that suggests that the roles of orexin receptor subtypes in depressive and anxiety-like behavior may be brain region-specific [12]. Interestingly, studies indicate that orexins may directly excite serotonin neurons by activating K+ leak currents or Na+-dependent nonselective cation channels (NSCCs) [30,31]. Furthermore, as shown by Liu and co-workers, orexins at higher concentrations indirectly inhibit serotonergic neurons by exciting GABA-ergic interneurons [32]. The systemic administration of SB-33487, an orexin receptor antagonist-1 (SORA1), in rats with CO_2_ and sodium lactate-induced panic silenced the orexin gene in the hypothalamus and blocked panic responses and anxiety-like behavior [12,19,33]. However, the intracerebroventricular injection of SB-334876 was found to decrease seizures and anxiety in rats treated with pentylenetetrazol [34]. Interestingly, the systemic administration of SORA1 did not influence anxiety-like behavior in the open field [35], whereas microinjection into the trigeminal nucleus caudalis reduced orofacial pain-induced anxiety in rats [36]. As stated in the reports of Johnson and co-workers, OXR2 maps the most to the histaminergic wake-promoting region, whereas OXR1 is more exclusive and denser in the anxiety and panic circuitry regions, such as the locus coeruleus, which suggests that it has a vital impact on mobilizing anxiety and panic responses [37]. There are also indications that ORX2 receptors mediate anxiolytic and antidepressant actions [38]. The authors conducted evaluations on mice using the stress alternatives model (SAM) and social interaction preference (SIP) test. It was stated that ORX2 activation reduced cued and conflict fear-conditioned freezing, reduced shock responses, increased attentiveness to escape routes, and promoted stress resilience. Other scientists also stated that anxiolysis was accompanied by the activation of some inhibitory neurons located in the amygdala. Mice, on the other hand, began escaping, which was reversed using ORX2 antagonists [38]. Therefore, these observations are not consistent with the mechanism of action of seltorexant, which is an ORX2 antagonist. As mentioned above, orexins are expressed differently depending on the brain region, which may be the reason for the discrepancy in the results.

**Figure 1:**
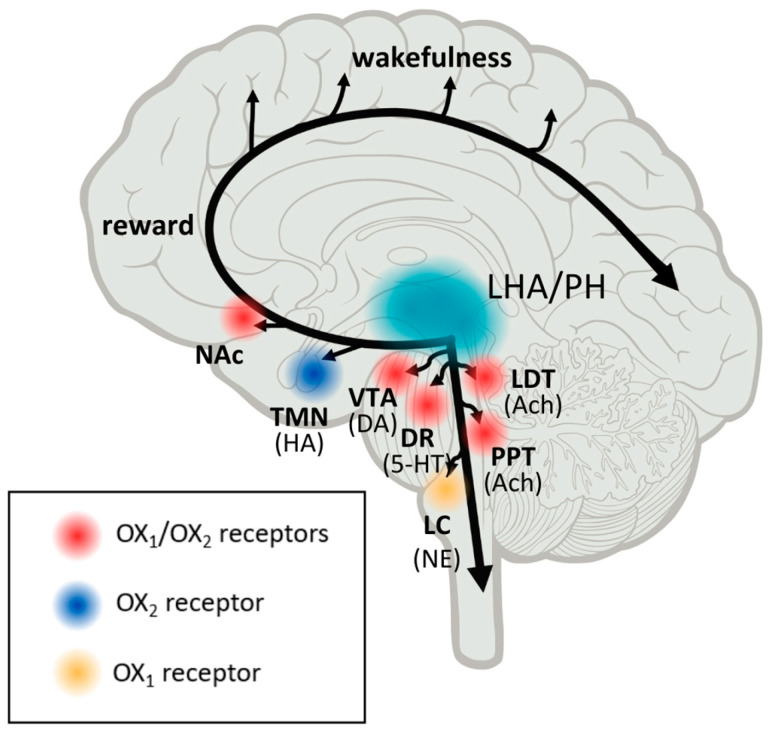
The OxR and orexin pathways linked to states of alertness, wakefulness, and pleasure. LHA, the lateral hypothalamic area; PH, posterior hypothalamus; NAc, nucleus accumbent; TMN, tuberomammillary nuclei; LDT, PPT, laterodorsal and pedunculopontine tegmental nuclei; LC, the locus ceruleus; DR, dorsal raphe nuclei; VTA, the ventral tegmental area; HA, histaminergic; DA, dopaminergic; ACh, cholinergic; NE, noradrenergic; 5-HT, serotonergic. Turquoise, orexinergic neuron projections; yellow, preferential OX1 receptor expression; blue, preferential OX2 receptor expression; red, both OX1 and OX2 receptor expression.

Thus, there is evidence linking the influence of the orexin system to the development of depression. Therefore, it is appropriate to search for new drugs that act on this system. An example of a drug that is under clinical trial is seltorexant, an OXR2 receptor antagonist (Figure 1).

## 4. Structure, Pharmacodynamics, and Pharmacokinetics of Seltorexant

Seltorexant, with the approved UPAC name [2-(4,6-dimethylpyrimidin-2-yl)-1,3,3a,4,6,6a-hexahydropyrrolo [3,4-c]pyrrol-5-yl]-[2-fluoro-6-(triazol-2-yl)phenyl]methanone, also known as JNJ-42847922, was developed as a potent selective antagonist of human OXR-2 for the treatment of insomnia as well as MDD.

**Figure 2:**
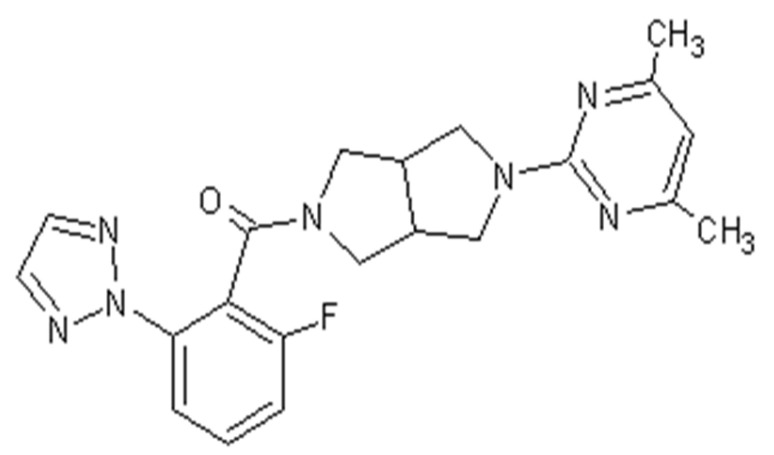
Chemical formula of seltorexant, a selective orexin antagonist.

**Figure 3:**
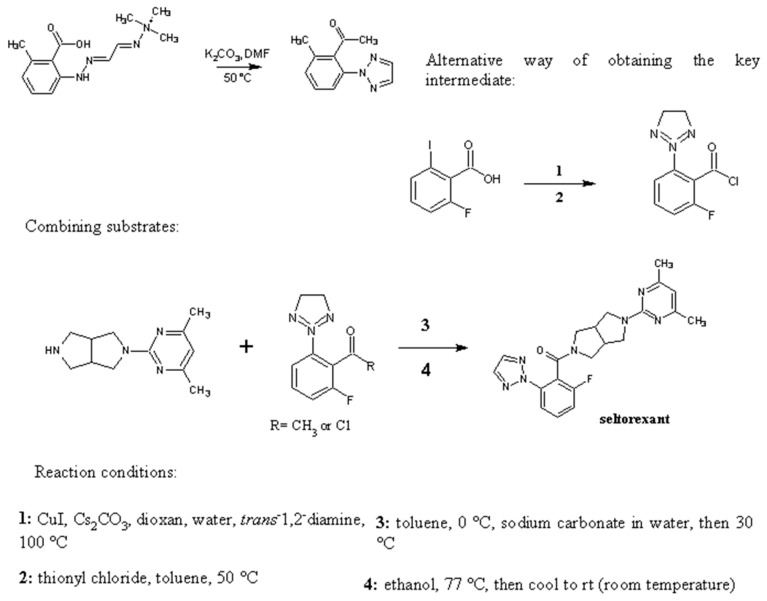
Possible methods of obtaining seltorexant, otherwise known as JNJ-42847922. Reactions adapted from Letavic et al., 2015 and Chen et al., 2020 [39,41].

In an original paper from 2015, a first optimized synthesis of seltorexant was described [39]. First, a transformation to 2-fluoro-6-[1,2,3]triazol2-yl-benzoic acid was performed in dioxane via a copper-mediated coupling process. In a separate reaction, 2-Benzyl-5-(4,6-dimethyl-pyrimidin-2-yl)-octahydro-pyrrolo [3,4-c]pyrrole was condensed with 2-chloro-4,6-methylpyrimidine, which was then hydrogenated in the presence of acetic acid to give the acetic acid salt. Benzoic acid was then transformed into acid chloride, which subsequently reacted to yield crude seltorexant. Recrystallization from ethanol resulted in high yields [39,40] (Figure 2 and Figure 3).

Another efficient method of the synthesis of 2-aryl-1,2,3-triazoles based on intramolecular N-N bond formation, presumably by the SN2 displacement of hydrazonium species in the presence of a base, has been established. The approach makes use of easily available starting materials, milder reaction conditions, and comprehensive region-selective control. The simplicity of the reaction sequence, wide substrate scope, and high efficiency of N,N-bond production is beneficial, as it allows for the quick and easy synthesis of the substrate needed to obtain seltorexant [41].

As the orexin system still is a relatively new discovery, many questions concerning the position of orexin receptors in the body and brain arise. Seltorexant was tested among previously known selective OX2R antagonists and found to have the potential to be an OX2R imaging probe. Bai and colleagues synthesized the labeling precursor and obtained [18F] Seltorexant with success. Ex vivo autoradiography investigations on mouse brain slices revealed that [18F] Seltorexant had a high binding affinity for OX2R. In vivo PET imaging in animals revealed that [18F] Seltorexant penetrated the blood–brain barrier (BBB) sufficiently for OX2R imaging in the brain. [18F] Seltorexant displayed good binding selectivity and specificity, according to regional brain biodistribution analysis and binging experiments. Pretreatment with the competitive P-gp inhibitor CsA increased [18F] Seltorexant brain uptake, implying that [18F] Seltorexant is a substrate of the efflux transporter. [18F] Seltorexant is a possible PET probe for OX2R imaging in the brain, which could aid in the development of future OX2R PET probes [42].

According to the comprehensive study conducted by Bonaventure and co-workers, seltorexant was tested for its selectivity in a panel of 50 different receptors, ion channels, and transporter assays as well. It was stated that seltorexant at the concentration of 1 mM was revealed to have no significant affinity for any receptor, transporter, or ion channel other than the OX2R. Additionally, the selectivity was confirmed in vitro on both human and rat OX2R by measuring changes in intracellular calcium in cell culture assays. These researchers claim that high affinity to OX2R was reflected in potent functional activity (Bonaventure et al., 2015). It is noteworthy that seltorexant crosses the BBB and binds with OX2R, which was confirmed in vivo in Sprague Dawley rats. The time and dose dependency of the occupancy of OX2R was also assessed through ex vivo receptor binding autoradiography of competitive radioligand binding assays using [3H]EMPA ([3H]N-ethyl-2-[(6-methoxy-pyridin-3-yl)-(toluene-2-sulfonyl)-amino]-N-pyridin-3-ylmethyl-acetamide (EMPA) in rat cortex tissue sections. The oral administration of seltorexant in a dosage of 30 mg/kg inhibited EMPA binding to the rat cortex, which proves sufficient oral bioavailability and brain penetration. The highest level of OX2R occupancy by seltorexant was noticed 60 min after administration (74.66%). However, after 4 h, the number of occupied receptors declined to 40%. After 24 h, scientists reported no occupancy of OX2R by seltorexant. Additionally, the receptor occupancy and plasma/brain concentration of the drug were correlated. When the number of occupied OX2R decreased, the concentration of seltorexant in plasma/brain increased. During the same experiment, it was stated that seltorexant indicated low OX1R occupancy [40].

With regard to the pharmacokinetic properties, Bonaventure and co-workers stated that the plasma pharmacokinetics of seltorexant were characterized by rapid absorption followed by an apparent monophasic decline, with the mean Tmax ranging from 0.33 to 0.5 h and a half-life of approximately 2 h. The Cmax and area under the curve values were dose-dependent, but changed in a dose-proportional manner [40]. The values are presented in Table 1.

## 5. Pre-Clinical Studies on Seltorexant

Due to the fact that seltorexant is an orexin receptor antagonist, it is appropriate that research on its antidepressive and hypnotic properties was conducted, especially given that insomnia is correlated with MDD. With the aim of better understanding seltorexant’s mechanism of action and its influence on organisms, a number of both human and animal studies were conducted.

In 2015, it was stated that seltorexant dose-dependently induced and prolonged sleep in Sprague Dawley rats [39,40]. The sleep–wake patterns were investigated during both the dark (active) phase and the light (rest) phase. In the study conducted by Letavic and colleagues in the active phase, rats were orally dosed at the beginning of the dark phase with 1, 3, 10, and 30 mg/kg. During the study conducted in the light phase by Bonaventure and co-workers, rats were orally administered 3, 10, and 30 mg/kg seltorexant after two hours in the light phase. As a result, these scientists stated that the administration of seltorexant produced a dose-dependent reduction in NREM sleep latency as well as an increase in NREM sleep time in the first 2 h, independently from the phase. An effective dose was estimated at 3 mg/kg both for sleep induction and sleep promotion. It is noteworthy that the dose–response effects were more apparent during the dark phase than the light phase. The scientists also indicated that the time spent in NREM sleep was still gradually prolonged from doses of 3 to 30 mg/kg. It was also stated that NREM sleep time in the light phase was increased with the highest tested dose (30 mg/kg) and was associated with the prolongation of the NREM bout duration with no change in the number of NREM bouts, which suggests enhanced sleep consolidation. Importantly, there was no significant effect on REM sleep duration and REM sleep latency at the tested doses. REM sleep latency was reduced, and REM sleep time increased only at the dose of 60 mg/kg tested during the dark phase. As a result, the scientists indicate that sleep architecture was preserved at all tested doses in the light phase. However, NREM and REM sleep, as well as the index of sleep intensity, were not altered and the hypnotic effect lasted for 2 h after drug administration during both the light and dark phase. It is noteworthy that the animals showed a reduction in locomotor activity and small decrease in body temperature. Importantly, the sleep promoting effects were maintained upon 7-day repeated dosing. During the same evaluation, the scientists stated that seltorexant had no effect on sleep parameters in mice lacking the OX2R, which also proves the selectivity of the compound [39,40].

During the same studies, the scientists evaluated whether or not the administration of seltorexant influences dopamine release in the rat nucleus accumbens. In vivo microdialysis was investigated in freely moving rats [40]. It transpired that seltorexant at the dose of 30 mg/kg had no effect on extracellular dopamine release. The results were compared with amphetamine as a positive control group. Furthermore, the study assessed whether the administration of seltorexant had an effect on place preference in mice after subchronic conditionings. This paradigm evaluates the rewarding effect of a psychoactive substance, measuring the time spent by an animal in a compartment where an addictive substance was present during conditioning. Evaluation was conducted in three groups. In the first, mice were treated with zolpidem (10 mg/kg), in the second, they treated with seltorexant (10 mg/kg), and in the third, with a vehicle using a conditioned place preference (CPP) test. Additionally, amphetamine (2 mg/kg) was also added as a positive control. The scientists stated that mice treated with zolpidem or/and amphetamine spent significantly more time in the drug-paired chamber during the place preference test than mice in the vehicle group. However, the results for the seltorexant-treated group did not differ significantly from the vehicle group, which proves that the use of seltorexant does not produce place preference, which suggests that seltorexant shows no addictive properties [40].

As has been acknowledged, hypothalamic–pituitary–adrenal (HPA) axis activity increases during stress. As the high activity of the HPA axis is considered as a depression factor, any influence on this pathway may have a potential application in the treatment of depression. Thus, as OX2R was found to be preferentially expressed in the paraventricular hypothalamic nucleus involved in the regulation of the HPA, Yun and co-workers conducted an evaluation of the influence of inhibition of OXR2 on stress-induced adrenocorticotropic hormone (ACTH) release in mice. The mice were stressed by changing cages, and serum ACTH levels were measured at 2 and 4 h of the light phase. Cage exchange induced stress, which resulted in a significant increase in ACTH in the wild-type mice. Interestingly, mice deprived of OX2R exhibited a blunted stress response. Importantly, there was no release of ACTH in rats treated with seltorexant (30 mg/kg). However, pre-treatment with SB-649868, which is a dual OX1R and OX2R antagonist, only partially weakened the ACTH release, which proves the predominant role of OX2R in the regulation of the HPA axis. During these studies, the scientists also evaluated whether or not the intrinsic and distinct sleep-promoting properties of each orexin receptor antagonist could account for the potential differential stress response. Thus, mice with implanted electrodes for EEG sleep recording were treated with seltorexant (30 mg/kg), SB-649868 (30 mg/kg), or vehicle. The administration of both compounds resulted in a reduction in the latency to non-rapid eye movement (NREM) sleep without affecting its duration, although a prevalent REM sleep-promoting effect was observed only in mice treated with SB-649868 [43]. Thus, the results indicate that seltorexant can be used in the treatment of hyperarousal insomnia characterized by overactivity of HPA, which is one of the pro-depressive factors. All presented data is presented in the Table 2. 

## 6. Clinical Studies on Seltorexant

De Boer and co-workers conducted human research on the influence of seltorexant in patients with insomnia without psychiatric disorders. The research had two outcomes. The main objective was to investigate the effect of seltorexant on sleep efficiency after single and multiple dosages. The secondary objectives referred to included the evaluation of total sleep time and latency to persistent sleep and wake after sleep onset. The duration of the study was divided into two periods. During period 1, patients received 40 mg of seltorexant for five days and during period 2, they received a placebo, or vice versa. Sleep parameters were measured using polysomnography over 8 h on day 1/2 (single dose) and on day 5/6 (multiple doses), and subjective sleep parameters were assessed using questionnaires. Twenty-seven patients completed the trial. It was noticed that the treatment with seltorexant resulted in a prolonged total sleep time, and shorter latency to persistent sleep and wake after sleep onset as well. Patients treated with seltorexant also had a persistent reduction in the time to REM onset as well as increase in the total duration of REM sleep [44], which confirms seltorexant as a promising candidate for the treatment of insomnia.

Hypnotic activity was also confirmed during human research conducted by van der Ark and co-workers, who stated that patients treated with seltorexant reported somnolence. During the study, dosage was determined. It was found that somnolence was inconsistent over time following the administration of a placebo and 5 mg dose. However, patients treated with seltorexant at the doses ≥ 10 mg reported daily somnolence, although somnolence of moderate intensity was reported at dose ≥ 20 mg [45].

Assuming that excessive arousal has a role in the pathophysiology of MDD, Recourt and co-workers conducted human research on the use of seltorexant in patients with MDD, during which they focused on the antidepressive and hypnotic activity of the compound. In this study, 47 patients who had a Depressive Symptomatology Inventory (IDS) score greater than or equal to 30 were enrolled, examining the effect of nocturnal arousal suppression on depressive symptoms. Additionally, depression symptoms were assessed using the 17-item Hamilton Depression Rating Scale (HDRS17). Effects on sleep were assessed using polysomnography and the Leeds Sleep Evaluation Questionnaire (LSEQ). As the researchers report, at baseline, the severity of depressive symptoms was correlated with sleep efficiency, wake after sleep onset (WASO), and the duration of stage 2 sleep and rumination. The patients treated with seltorexant at a dose of 20 mg daily for 10 days exhibited an improvement in core depressive symptoms compared to the placebo group and group treated with diphenhydramine. It also affected core symptoms of depression without changes in the hypnogram. Additionally, the antidepressant activity of seltorexant coincided with a growth in EEG power and a relative increase in delta and decrease in theta, alpha, and beta power during stage 2 sleep. It is noteworthy that the antidepressant activity of seltorexant was maintained with continued treatment up to 28 days [46].

In 2021, Slavitz et al. [47] published the results of a study conducted on patients with MDD who had an inadequate response to one to three selective serotonin/serotonin-norepinephrine reuptake inhibitors in the current episode. The recruited patients were required to have a Montgomery–Åsberg Depression Rating Scale (MDRS) score ≥ 25 at screening, and showed no improvement in MADRS total score from the screening to baseline visit better than 20%. Additionally, patients were stratified by baseline Insomnia Severity Index (ISI) scores (ISI ≥ 15 vs. <15). As researchers report, the purpose of the study was to replicate and extend previous observations. Additionally, the study was aimed at finding an effective dose of seltorexant. During the trial, patients were randomized to receive 10, 20, or 40 mg of seltorexant or placebo once daily adjunctively to antidepressants currently taken. Interestingly, after a period of 6 weeks, newly recruited patients received only 10 or 20 mg of seltorexant or placebo. The 40 mg dose was no longer assigned. The primary outcome of the evaluation was a change from baseline MDRS total score at week 6. The results of the study confirmed the antidepressive properties of seltorexant. Moreover, the analysis showed a greater improvement in MADRS total score in the seltorexant 20 mg group than in the placebo group at both week 3 and 6. Interestingly, the greater improvement at week 6 was noticed in patients who had had baseline Insomnia Severity Index (ISI) scores ≥ 15 vs. those with ISI < 15 [47]. All data is presented in the Table 2.

## 7. Safety of Seltorexant

During every clinical study, it is crucial to meticulously monitor every unwanted drug effect as well as possible interactions. The influence of seltorexant on motor coordination and alcohol-induced ataxia was investigated by Bonaventure and co-workers in rats. Their study with the use of rotarod apparatus revealed that seltorexant had no effect on motor coordination in rats at a dose that induced sleep. The compound did not modify the ataxia effect of alcohol either. For comparison, zolpidem intensified the effect of alcohol. As the researchers claim, it suggests that seltorexant did not have any myorelaxant effect. Additionally, scientists conducted toxicological studies of the compound, which lasted up to one month and was conducted in both rats and dogs. As a result, the scientists indicated that seltorexant has a suitable safety profile and can be allowed for human studies. According to the research, seltorexant was well tolerated in a single dose as well as multiple dosages. It also did not indicate genotoxicity. Furthermore, it was well tolerated in a dog cardiovascular safety study [40].

In a meta-analysis from 2022 of all gathered data, a group of researchers aimed to assess efficacy (measured as patient-rated quality of sleep or satisfaction with sleep index), all causes of discontinuation (the proportion of patients who stopped treatment for any reason, which is used as a measure for the acceptability of treatments because it encompasses both efficacy and tolerability), tolerability (treatment discontinuation measured by the proportion of patients who withdrew due to any ailment), and tolerability (treatment discontinuation measured by the proportion of patients suffering from at least one adverse effect). Seltorexant had fewer side effects than benzodiazepines, eszopiclone, zolpidem, and zopiclone at the end of the study. Seltorexant was mentioned as a well-tolerated drug, but data on efficacy and other important outcomes were limited, making firm conclusions impossible [48].

In 2021, a randomized, placebo-controlled, adaptive dose-finding trial that looked at the efficacy and safety of three doses of seltorexant (10, 20, and 40 mg) as an adjunctive therapy in patients with MDD who had not responded well to previous antidepressant treatment was published. Patients were divided into two groups based on their location and level of sleep disturbance (Insomnia Severity Index (ISI) score 15 vs. 15). Overall, 55/146 (37.7%) patients in the seltorexant groups (970, 10 mg: 11/33 (33.3%), 20 mg: 25/61 (41.0%), and 40 mg: 19/52 [36.5%]) and 56/137 (40.9%) patients in the placebo group experienced at least one TEAE (treatment-emergent adverse event). Headaches (9 (6.2%)vs. 9 (6.6%)), somnolence (9 (6.2%) vs. 7 (5.1%)), and nausea (8 (5.5%) vs. 4 (2.9%)) were the most common TEAEs (5% of patients in the seltorexant groups vs. placebo). Overall, 7 (5.1%) patients in the placebo group and 12 (8.2%) patients in the seltorexant treatment group, which includes 7 (11.5%) patients in the seltorexant 20 mg treatment subgroup, experienced somnolence-related adverse events [47].

In the seltorexant-treated groups, the most common TEAEs that led to discontinuation were insomnia (2.1%), sleep paralysis (1.4%), irritability, nausea, vomiting, and increased alanine aminotransferase and aspartate aminotransferase (0.7% each). The majority of TEAEs were mild to moderate in severity. No deaths or serious TEAEs were reported in any of the seltorexant treatment groups. One patient in the placebo group experienced a serious polycythemia vera TEAE. The most common TEAEs reported in the seltorexant treatment groups compared to placebo were abnormal dreams (2.7% vs. 0.7%), sleep paralysis (1.4% vs. 0.7%), and nightmares (1.4% vs. 0) [47].

However, no clear evidence of a dose effect on the reporting of TEAEs of special interest was found when the seltorexant 10 mg, 20 mg, and 40 mg doses were compared. There was no clinically significant increase in suicidal ideation across treatment groups, and no case of suicidal behavior was reported based on the reporting of such a serious adverse event or the change in C-SSRS score [47].

In the 2018 study [45], no deaths or serious adverse events were reported. All 30 subjects in the active treatment group (taking seltorexant, otherwise known as JNJ-42847922) and 5/10 subjects (50.0%) in the placebo group experienced at least one TEAE. On day 3 and 4, one subject receiving 60 mg JNJ-42847922 reported insomnia at night and somnolence during the day, and withdrew consent, so the study was terminated on day 5. Nervous system disorders, somnolence, headaches, dizziness, nausea, and other gastrointestinal disorders were the most frequently reported TEAEs, with an incidence greater than 5% in subjects receiving JNJ-42847922 or placebo. There were no consistent dose-related changes in mean vital signs (heart rate and blood pressure) or mean ECG parameters (PR, QRS, QT, QTcB, and QTcF intervals) from baseline. The C-SSRS (the Columbia Suicide Severity Rating Scale) and clinical laboratory data showed no significant abnormalities [45].

The effect of seltorexant in both medicated and antidepressant-naive patients with MDD in a current major depressive episode of moderate severity was investigated in a 2019 exploratory safety, tolerability, and efficacy phase 1b study. TEAEs were reported separately for women of child-bearing potential (WOCBP) and males/postmenopausal and surgically sterilized women, who received study medication for 11 and 28 days, respectively. Following placebo, seltorexant, and diphenhydramine treatment, 4/6, 8/10, and 2/6 subjects in the WOCBP group, respectively, reported TEAEs [46].

In male and female subjects who received study medication for 28 days, these numbers were, respectively, 3/6, 8/12, and 6/7 for the placebo, seltorexant, and diphenhydramine. In the WOCBP group, all TEAEs were reported by a single subject, whereas somnolence and nasopharyngitis were reported by three and two subjects who received seltorexant, respectively, in the remaining subject group. The majority of the TEAEs were mild to moderate in severity, and they were all transient in nature [46].

After the study, one serious adverse event (SAE) was reported; one subject randomized to the diphenhydramine group committed suicide. During the study, no other SAEs occurred [46].

## 8. Conclusions

Orexins were initially described as feeding behavior regulators. However, they had been found to be involved in various processes such as the coordination of emotion, energy homeostasis, reward, and arousal as well. There is evidence for the relationship between a dysregulated orexin system and mental disorders. As was acknowledged, orexins project to stress-sensitive brain regions. Thus, if stress (as the one of the prominent depression and insomnia causing factors) is correlated with orexin system functionality, it means that orexins can be new promising targets for the treatment of MDD and insomnia as well. Seltorexant, which was described as an orexin 2 receptor antagonist, was comprehensively tested for its selectivity and activity in both human and animal studies. The results indicate that seltorexant promotes and prolongs total sleep time. It also shortens latency to persistent sleep as well as wake after sleep onset. Importantly, it preserves sleep architecture and can be used in a treatment of hyperarousal insomnia characterized by overactivity of the HPA. Furthermore, antidepressant activity was confirmed to be maintained up to 28 days with continued treatment. Additionally, the co-administration of seltorexant with other antidepressant treatments improved the state of patients, and an effective dose was estimated at 20 mg. The use of seltorexant is considered safe as studies confirmed that it does not indicate genotoxicity. It was acknowledged that it was well tolerated during a dog cardiovascular safety study. Comprehensive studies also indicated that seltorexant had fewer side effects than benzodiazepines, eszopiclone, zolpidem, and zopiclone. Observed side effects were headaches, somnolence, dizziness, nausea, and vomiting. It is noteworthy that there was no clinically significant increase in suicidal ideation across treatment groups, and no case of suicidal behavior. In addition, studies did not indicate consistent dose-related changes in mean vital signs (heart rate and blood pressure) or mean ECG parameters from baseline. To summarize, there are indications that seltorexant is a promising candidate for the treatment of depression and insomnia. Research shows that insights on the orexin system can provide a new approach for the treatment of mental conditions. Thus, studies on the orexin system and its ligands should be continued.

## Figures and Tables

**Table 1:** Pharmacokinetics from plasma of healthy male subjects after single oral doses (varying dosage) of seltorexant.

Dose [mg]	10 mg	20 mg	40 mg	80 mg
Cmax [ng/mL]	309 ± 74	556 ± 104	743 ± 149	1208 ± 291
Tmax [h]	0.33	0.42	0.42	0.50
AUCinf [ng.h/mL]	802 ± 275	1616 ± 607	1807 ± 533	3655 ± 1064
t^½^ [h]	2.02 ± 0.26	2.11 ± 0.47	2.02 ± 0.30	2.44 ± 0.46

**Table 2:** Summary of preclinical studies and clinical trials, both in in vivo and animal models, performed in order to assess the pharmacodynamic and pharmacokinetic characteristics of seltorexant.

Animal Model Assays of Pharmacodynamic and Pharmacokinetic Characteristics of Seltorexant			
Publication	Study Design	Objective of Study	Results and Clinical Implications
Letavic et al. (2015) [39]	Structure–activity relationship (SAR) study	Stability in liver microsomes assay; Pharmacokinetic studies assay; Brain tissue binding assay; Plasma protein binding assay; Caco-2 permeability assay; CYP Inhibition Assay.	Seltorexant showed acceptable bioavailability in vivo. Seltorexant has relatively high free fractions as measured by protein binding in rats and humans, and it exhibits a high free fraction in rat brain tissue. It also shows moderate CYP inhibition. It dose-dependently occupies the OX2R and the EC50 for occupancy in this experiment with 171 ng/mL, which corresponds to an oral ED50 of 3 mg/kg. Oral administration of seltorexant at the onset of the dark phase produced a dose-dependent reduction in NREM latency and an increase in NREM sleep time with no significant effect on REM sleep. Seltorexant was shown to be a promising compound in clinical trials for treatment for insomnia.
Bonaventure et al. (2015) [40]	Randomized, double-blind, placebo-controlled phase 1 trial; double-blind, randomized, parallel-group phase 2a trial	*Phase 1:* single oral dose of JNJ-42847922 (ranging from 1 to 30 mg) or placebo. *Phase 2:* 5 or 10 mg of seltorexant or placebo once daily for 4 weeks.	Seltorexant (JNJ-42847922) is a high-affinity selective OX2R antagonist. After oral administration, it crosses the blood–brain barrier and binds to the OX2R in the rat brain. It induces and prolongs sleep in rats in a dose-dependent manner. In rats, sleep-promoting effects are maintained after 7 days of repeated dosing. In mice lacking the OX2R, it has no effect on sleep parameters. JNJ-42847922 has no effect on dopamine release in the rat nucleus accumbens and produces no place preference in mice following subchronic conditioning. It has no effect on rats’ motor coordination or alcohol-induced ataxia. In a first-in-human trial, it demonstrated a suitable pharmacokinetic profile and promoted somnolence.
Yun et al. (2017) [43]	Animal assay	Intracerebroventricular (ICV) injection of JNJ-42847922 (Seltorexant). Induction of stress through exposure to an elevated platform for 150 min.	After ICV administration of JNJ-42847922, a lack of stress-induced increase in ACTH levels in an elevated platform test was noted. Seltorexant inhibits the stress-induced c-Fos expression in the paraventricular nucleus of the hypothalamus.
Clinical trials of seltorexant			
De Boer et al. (2018) [44]	Randomized, double-blind, placebo-controlled, parallel-group, dose-ranging phase 2 study	A total of 27 participants were randomly assigned to receive seltorexant (5 mg, 10 mg, 20 mg, or 40 mg) or placebo for 4 weeks.	The mean differences in sleep efficiency (% (SD)) between seltorexant and placebo at day 1/2 were 5.8 (9.2) and 7.9 (9.8), respectively (p 0.001 at both time points), in total sleep time (min (SD)) 27.7 (44.3) and 37.9 (47.1), respectively, in latency to persistent sleep (min (SD)) −18.8 (21.3) and −29.9 (27.7), respectively, and in wake after sleep onset (46.5). Headache and somnolence were the most common side effects. Treatment with seltorexant resulted in a prolonged total sleep time, and shorter latency to persistent sleep and wake after sleep onset.
Van der Ark et al. (2019) [45]	Randomized, double-blind, placebo-controlled crossover study	Five consecutive cohorts of healthy subjects received multiple daily doses of JNJ-42847922 (5 mg or 10 mg) or placebo for 4 days.	After the morning dose, JNJ-42847922 was rapidly absorbed. The median Tmax was 0.5–1.5 h, and the mean t1/2 was 2–3 h. At 20 mg dose levels of JNJ-42847922, mean Cmax and mean area under the curve increased less than dose-proportionally. JNJ-42847922 consistently induced somnolence at doses of 20 mg on all study days. Multiple daytime administration of seltorexant induced somnolence in healthy subjects without any residual effects on central functions.
Recourt et al. (2019) [46]	Double-blind, randomized, placebo-controlled study	A total of 47 MDD patients with a total Inventory of Depressive Symptomatology (IDS) score of ≥30 at screening were included. Participants were randomized to receive either seltorexant or placebo for 8 weeks. The starting dose was 20 mg/day and was increased to 40 mg/day after 1 week.	Ten days of treatment with seltorexant resulted in a significant improvement in core depressive symptoms compared to placebo; the antidepressant efficacy of seltorexant was maintained with continued treatment up to 28 days. Seltorexant showed a statistically significant improvement in MADRS total score compared to placebo.
Savitz et al. (2021) [47]	A phase 2b, randomized, placebo-controlled, adaptive dose-finding study	Patients were randomized to receive either seltorexant (5 mg, 10 mg, or 20 mg) or placebo once daily for 8 weeks as adjunctive therapy to their current antidepressant treatment.	Seltorexant at all doses resulted in statistically significant improvements in MADRS total score compared to placebo. Significant improvements were observed in HAM-D total score, CGI-S score, sleep quality VAS score, and ISI score for seltorexant compared to placebo.

Explanation of used abbreviations: CL (mL/min/kg)—clearance; Vss (L/kg)—volume of distribution at steady state; t^1/2^ (h)—i.v. half-life; Rat BB—rat brain tissue binding; RLM stability—stability in rat liver microsomes. Data reported as extraction ratio; HLM stability—stability in human liver microsomes. Data reported as extraction ratio.

## Data Availability

Not applicable.
